# Case Report: Variety of Target Antigens During 1 Year Follow-Up of a Patient Initially Diagnosed With Bullous Pemphigoid

**DOI:** 10.3389/fimmu.2021.825226

**Published:** 2022-01-13

**Authors:** Hua Qian, Zhijun Zhou, Luhuai Shi, Huicheng Li, Weijun Liu, Yong Ai, Yangmin Gao, Suying Feng, Takashi Hashimoto, Xiaoguang Li

**Affiliations:** ^1^ Dermatology Hospital of Jiangxi Province, Jiangxi Provincial Clinical Research Center for Skin Diseases, Candidate Branch of National Clinical Research Center for Skin Diseases, Dermatology Institute of Jiangxi Province, The Affiliated Dermatology Hospital of Nanchang University, Nanchang, China; ^2^ Institute of Dermatology, Chinese Academy of Medical Sciences and Peking Union Medical College, Nanjing, China; ^3^ Department of Dermatology, Osaka City University Graduate School of Medicine, Osaka, Japan

**Keywords:** bullous pemphigoid, autoimmune bullous disease, mucosal-dominant-type pemphigus vulgaris, autoantibody, epitope spreading

## Abstract

Autoimmune bullous diseases (AIBDs), presenting cutaneous and/or mucosal bullous lesions, are classified into pemphigus and pemphigoid diseases. A longtime observation for complicated AIBD cases is rarely reported. In this study, serum samples of one AIBD patient were collected at seven different time points during the disease course including a relapse, which were examined by our conventional and newly developed methods for the detection of autoantibodies. Interestingly, we found changes of both the presence and the titers of various autoantibodies in accordance with the changes of clinical features during the whole disease course, which indicated that the patient started as bullous pemphigoid and relapsed as concurrence of bullous pemphigoid and mucosal-dominant-type pemphigus vulgaris.

## Introduction

Autoimmune bullous diseases (AIBDs) clinically show mucocutaneous lesions and are classified into pemphigus and pemphigoid diseases (1). Two major pemphigus diseases are pemphigus vulgaris showing antibodies to desmoglein (Dsg) 3 and/or Dsg1 and pemphigus foliaceus showing anti-Dsg1 autoantibodies (2). Pemphigus vulgaris with only anti-Dsg3 autoantibodies is defined as mucosal-dominant-type pemphigus vulgaris, while pemphigus vulgaris with both mucosal and cutaneous lesions is diagnosed as mucocutaneous-type pemphigus vulgaris (3). Pemphigoid diseases are classified into various subtypes according to the clinical features and differences of autoantibodies (4). Bullous pemphigoid, the most common pemphigoid subtype, shows autoantibodies against BP180 and/or BP230 and presents mainly cutaneous lesions (5).

Increasing diversity of autoantibodies is usually explained by “B-cell epitope spreading” which is an antigen-driven process and leads to diversification of the epitopes recognized by the immune system and thus to maturation of the humoral response (6). Epitope spreading has been reported in various AIBDs (7, 8).

## Case Description

A 61-year-old female patient, presenting with widely distributed skin lesions, was diagnosed as bullous pemphigoid based on histopathological findings of subepidermal blister and infiltration of lymphocytes and eosinophils and linear IgG and C3 depositions in the basement membrane zone (BMZ) in direct immunofluorescence. This case showed a recurrence with mucocutaneous lesions 270 days later. Details for changes of clinical features at various time points, including a relapse at day 270, as well as changes of steroid doses used, are shown in **Table 1** and **Table S1**.

**Table 1:** Clinical features of the patient during the disease course.

Clinical features/days	On the skin					On the oral mucosa		Methylprednisolone dose (mg/day)
	Blister	Erythema	Erosion	Crust	Itching	Blister	Erosion	
0	**+**	**+**	**+**	**+**	**+**	−	−	30
7	−	−	−	−	−	−	−	28
55	−	−	−	−	−	−	−	22
105	−	−	−	−	−	−	−	14
120	−	−	−	−	−	−	−	12
270	**+**	**+**	**+**	**+**	**+**	**+**	**+**	Stopped from day 150 and restarted with 40 mg/day from day 270
279	−	−	−	−	−	−	−	38

**+**, positive; −, negative.

The serum samples of this patient were collected at seven different time points during the disease course for our autoantibody analyses with various conventional and newly developed methods. This study was performed according to the Declaration of Helsinki and approved by the Ethics Committee of Dermatology Hospital of Jiangxi Province. Informed consent of this patient was obtained.

**Figure 1:**
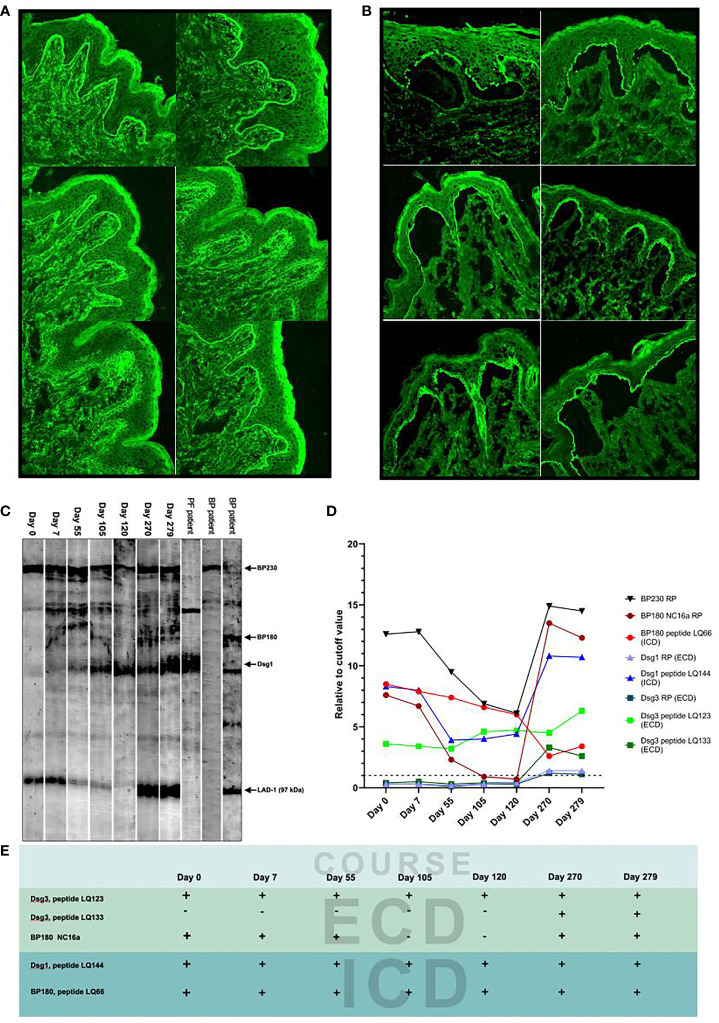
Detection of IgG autoantibodies by various methods. **(A)** The results of indirect immunofluorescence (IIF) for the seven patient sera collected at indicated days. **(B)** The results of indirect immunofluorescence using salt-split skin (ssIIF) for the seven patient sera collected at indicated days. The IIF and ssIIF results of day 270 were similar to those of day 279 and therefore not shown. In both IIF and ssIIF, staining to cytoplasm but not to cell surfaces in the lower epidermis was seen at days 105 and 120, while clear cell surface staining in the lower epidermis was found only at days 270 and 279. Reactivity with BMZ was also negative at days 105 and 120 in both IIF and ssIIF. The upper, middle and lower images (from left to right) are days 0 and 7, 55 and 105, and 120 and 279, respectively. **(C)** The results of immunoblotting of normal human epidermal extract for the seven patient sera. The sera of pemphigus foliaceus and bullous pemphigoid patients were used as positive controls to indicate the protein bands for BP230, BP180, Dsg1, and the 97-kDa LAD-1, a truncated form of BP180. None of the seven patient sera reacted with intact BP180 and Dsg3. **(D)** The results of ELISAs using various recombinant proteins and peptides. Dot line indicates cutoff value. **(E)** Summary of the reactivity with major peptides and recombinant protein of the patient sera during the disease course.

By indirect immunofluorescence using normal human skin (IIF), positive IgG reactivity with BMZ was found in sera at days 0, 7, 55, 270, and 279 and that with keratinocyte surfaces at the lowermost epidermis at days 270 and 279 (**Figure 1A**). By indirect immunofluorescence using salt-split skin (ssIIF), positive IgG reactivities with the epidermal side of the split skin and with cell surfaces in the lowermost epidermis were found in a similar manner to those found in IIF (**Figure 1B**). The findings in IIF and ssIIF at days 270 and 279 were almost the same, and therefore, the results at day 270 are not shown in **Figures 1A, B**.

By immunoblotting of normal human epidermal extract, BP230, Dsg1, and the 97-kDa LAD-1, a truncated form of BP180, were detected in all seven sera (**Figure 1C**).

Next, we performed ELISAs using various recombinant proteins (RPs) and peptides. ELISA kits (MBL, Nagoya, Japan) of BP180 NC16a domain, BP230, Dsg1, and Dsg3 were performed according to the instructions.

Autoantigen peptides were designed as reported previously (9), including 29 peptides covering full-length BP180, 20 peptides covering full-length Dsg3, and 25 peptides covering full-length Dsg1. Detailed information for peptides used is shown in **Table 2**. ELISAs with these peptides were performed as described previously (9).

**Table 2:** Information of peptides used in this study.

Autoantigens	Peptide name	Amino acid sequences	Starting amino acid position	ECD or ICD
BP180	LQ66	KTVSTKGKTTTADIC	411	ICD
Dsg1	LQ144	CGAPRSAAGFEPVPE	575	ICD
Dsg3	LQ123	CSPGTRYGRPHSGRL	603	ECD
Dsg3	LQ133	CNVREGIAFRPASKT	376	ECD

Dsg1, desmoglein 1; Dsg3, desmoglein 3; ECD, extracellular domain; ICD, intracellular domain.

The changes of titers of autoantibodies to BP230 RP, BP180 NC16a domain RP, and Dsg1 peptide LQ144 (intracellular domain, ICD) matched well to the disease course of this patient, while the changes of titers of autoantibodies to BP180 peptide LQ66 (ICD) and Dsg3 peptide LQ123 (extracellular domain, ECD) did not correlate with the disease course (**Figure 1D**). The changes of titers of autoantibodies to Dsg1 RP (ECD), Dsg3 RP (ECD), and Dsg3 peptide LQ133 (ECD) reflected the disease relapse (**Figure 1D**). Autoantibodies against Dsg3 peptide LQ133 (ECD) became positive after relapse probably *via* epitope spreading (**Figure 1E**).

Other tests, which are routinely used in our laboratory for the detection of other known AIBD autoantibodies (9–15), showed negative results for all the seven sera.

Concerning the therapies, at disease occurrence (day 0), methylprednisolone 30 mg/day was administrated, and then the dose was gradually reduced to 12 mg/day at day 120 with disease improvement (**Table 1**). However, the patient by herself stopped the methylprednisolone administration at day 150. At day 270, the disease relapsed, and the patient restarted methylprednisolone 40 mg/day (**Table 1**). At day 279, the methylprednisolone dose was decreased to 38 mg/day and then no new lesions appeared either on the skin or in the oral cavity (**Table 1**).

## Discussion

From the points of both clinical and immunological views, this case was considered to start as bullous pemphigoid because of IgG reactivity with BMZ and higher titers of autoantibodies against BP180 and BP230 and to relapse as concurrence of bullous pemphigoid and mucosal-dominant-type pemphigus vulgaris, because of IgG reactivity with cell surfaces in the lowermost epidermis (localization of Dsg3), increased titers of autoantibodies against Dsg3, and newly appeared oral mucosal lesions at relapse.

It is known that there is a subgroup of bullous pemphigoid patients who present with not only cutaneous lesions but also mucosal lesions, with oral mucosa, the most frequently affected mucosa (16–18). Oral mucosal lesions were considered to be related with anti-BP180 antibodies in bullous pemphigoid (16). In our patient, oral lesions only appeared after relapse, and BP180 epitopes recognized by serum autoantibodies were not changed at relapse, suggesting that autoantibodies against BP180 might not cause the newly appeared oral lesions after relapse. Autoantibody to Dsg3 peptide LQ133 (ECD) might contribute to the oral lesions because its titers raised at relapse. Although the reactivity with Dsg1 peptide LQ144 (ICD) also increased at relapse, its significance is currently unknown. Therefore, in our patient, pathogenic autoantigens were considered as BP180 NC16a domain at occurrence and BP180 NC16a domain and Dsg3 ECD at relapse. The results of this study also imply that detailed studies for autoantibodies are necessary in bullous pemphigoid patients with mucosal lesions.

In bullous pemphigoid, serum anti-BP180 antibody titers are related to disease activity and are useful for the evaluation of disease severity and the effectiveness of treatments (19, 20). However, this conclusion was mainly drawn from the results of studies of patients examined only at one time point, but not of patients examined at multiple time points. In our case, in which we examined the sera at several time points, anti-BP180 NC16a antibody titers decreased clearly with steroid treatment and corresponded well with disease progression, with increase at relapse and decrease after the second steroid treatment, supporting that serum anti-BP180 antibodies could reflect disease severity and the effectiveness of treatments. Anti-BP230 antibodies in our patient also reflected disease severity and the effectiveness of treatments, although previous studies reported that anti-BP230 antibody titers do not correlate with disease severity (19). In addition, the titers of anti-BP180 peptide LQ66 (ICD) did not correlate with disease progression in our patient. Similarly, the titers of autoantibodies to Dsg1 RP (ECD), Dsg3 RP (ECD), and Dsg3 peptide LQ133 (ECD), but not Dsg3 peptide LQ123 (ECD), reflected the disease relapse. These complex results in our study suggest that further evaluation of the relationship between autoantibodies and disease severity is necessary.

There are reports of patients developing both pemphigus and bullous pemphigoid. Some patients shift from one disease to another, as seen in our case, and others present dual serologic evidence (21–23). Epitope spreading was considered to be involved in these cases (7, 8, 21). In our patient, autoantibodies against Dsg3 peptide LQ133 (ECD) became clearly positive at relapse. Therefore, the autoantibodies against Dsg3 peptide LQ133 might be produced by epitope spreading.

The first methylprednisolone administration at 30 mg/day gradually decreased due to good treatment outcome. Although sudden withdrawal of methylprednisolone led to the disease relapse with more complicated situation, i.e., concurrence of bullous pemphigoid and mucosal-dominant pemphigus vulgaris, readministration of methylprednisolone at 40 mg/day was also effective.

For complicated AIBD cases, commercially available ELISAs may not be enough, and precise understanding of their pathogenicity needs more sophisticated tests, including the ELISAs of autoantigen peptides used in this study.

## Data Availability Statement

The original contributions presented in the study are included in the article/**Supplementary Material**. Further inquiries can be directed to the corresponding authors.

## Ethics Statement

The studies involving human participants were reviewed and approved by the Ethics Committee of Dermatology Hospital of Jiangxi Province. The patient/participant provided her written informed consent to participate in this study.

## Author Contributions

All authors were involved in drafting the article or revising it critically for important intellectual content, and all authors approved the final version to be published. HQ, XL, and TH had full access to all data in the study and took responsibility for the integrity of the data and the accuracy of the data analysis. TH and XL conceived and designed the project. HQ, ZZ, LS, HL, WL, YA, YG, SF, TH, and XL collected case information and laboratory data and analyzed the data.

## Conflict of Interest

The authors declare that the research was conducted in the absence of any commercial or financial relationships that could be construed as a potential conflict of interest.

## Publisher’s Note

All claims expressed in this article are solely those of the authors and do not necessarily represent those of their affiliated organizations, or those of the publisher, the editors and the reviewers. Any product that may be evaluated in this article, or claim that may be made by its manufacturer, is not guaranteed or endorsed by the publisher.

## References

[1] Egami S Yamagami J Amagai M. Autoimmune Bullous Skin Diseases, Pemphigus and Pemphigoid. J Allergy Clin Immunol (2020) 145(4):1031–47. doi: 10.1016/j.jaci.2020.02.013 32272980

[2] Schmidt E Kasperkiewicz M Joly P. Pemphigus. Lancet (2019) 394(10201):882–94. doi: 10.1016/S0140-6736(19)31778-7 31498102

[3] Hashimoto T. Recent Advances in the Study of the Pathophysiology of Pemphigus. Arch Dermatol Res (2003) 295 Suppl 1:S2–11. doi: 10.1007/s00403-002-0366-3 12677426

[4] Schmidt E Zillikens D. Pemphigoid Diseases. Lancet (2013) 381(9863):320–32. doi: 10.1016/S0140-6736(12)61140-4 23237497

[5] Bernard P Antonicelli F. Bullous Pemphigoid: A Review of its Diagnosis, Associations and Treatment. Am J Clin Dermatol (2017) 18(4):513–28. doi: 10.1007/s40257-017-0264-2 28247089

[6] Cornaby C Gibbson L Mayhew V Sloan CS Welling A Poole BD. B Cell Epitope Spreading: Mechanisms and Contribution to Autoimmune Diseases. Immunol Lett (2015) 163:56–68. doi: 10.1016/j.imlet.2014.11.001 25445494

[7] Holtsche MM Goletz S von Georg A van Beek N Hübner F Pigors M. Serologic Characterization of Anti- P200 Pemphigoid: Epitope Spreading as a Common Phenomenon. J Am Acad Dermatol (2021) 84(4):1155–7. doi: 10.1016/j.jaad.2020.07.076 32711089

[8] Schauer F Steinke H Thoma K Kiritsi D. Transition From Bullous Pemphigoid to Pemphigus Foliaceus: Intermolecular Epitope Spreading Thirteen Years After Initial Diagnosis. Acta Derm Venereol (2019) 99(11):1029–30. doi: 10.2340/00015555-3250 31282977

[9] Qian H Cao Y Sun J Zu J Ma L Zhou H. Anti-Human Serum Albumin Autoantibody May be Involved in the Pathogenesis of Autoimmune Bullous Skin Diseases. FASEB J (2020) 34(6):8574–95. doi: 10.1096/fj.201903247RR 32369236

[10] Hashimoto T Jin Z Ishii N. Clinical and Immunological Studies for 105 Japanese Seropositive Patients of Epidermolysis Bullosa Acquisita Examined at Kurume University. Expert Rev Clin Immunol (2016) 12(8):895–902. doi: 10.1080/1744666X.2016.1196136 27247994

[11] Dainichi T Kurono S Ohyama B Ishii N Sanzen N Hayashi M. Anti-Laminin Gamma-1 Pemphigoid. Proc Natl Acad Sci USA (2009) 106(8):2800–5. doi: 10.1073/pnas.0809230106 PMC2650346 19196964

[12] Li X Qian H Ishii N Yamaya M Fukuda H Mukai H. A Case of Concurrent Antilaminin Gamma1 Pemphigoid and Antilaminin-332-Type Mucous Membrane Pemphigoid. Br J Dermatol (2014) 171(5):1257–9. doi: 10.1111/bjd.13107 25262782

[13] Qian H Natsuaki Y Koga H Kawakami T Tateishi C Tsuruta D. The Second Study of Clinical and Immunological Findings in Anti-Laminin 332-Type Mucous Membrane Pemphigoid Examined at Kurume University - Diagnosis Criteria Suggested by Summary of 133 Cases. Front Immunol (2021) 12:771766. doi: 10.3389/fimmu.2021.771766 34899722 PMC8660687

[14] Chiorean R Danescu S Virtic O Mustafa MB Baican A Lischka A. Molecular Diagnosis of Anti-Laminin 332 (Epiligrin) Mucous Membrane Pemphigoid. Orphanet J Rare Dis (2018) 13(1):111. doi: 10.1186/s13023-018-0855-x 29980216 PMC6035451

[15] Li X Qian H Sogame R Hirako Y Tsuruta D Ishii N. Integrin β4 Is a Major Target Antigen in Pure Ocular Mucous Membrane Pemphigoid. Eur J Dermatol (2016) 26(3):247–53. doi: 10.1684/ejd.2016.2772 27193492

[16] Tanaka M Hashimoto T Dykes PJ Nishikawa T. Clinical Manifestations in 100 Japanese Bullous Pemphigoid Cases in Relation to Autoantigen Profiles. Clin Exp Dermatol (1996) 21(1):23–7. doi: 10.1111/j.1365-2230.1996.tb00006.x 8689764

[17] Ständer S Schmidt E Zillikens D Ludwig RJ Kridin K. Immunological Features and Factors Associated With Mucocutaneous Bullous Pemphigoid - a Retrospective Cohort Study. J Dtsch Dermatol Ges (2021) 19(9):1289–95. doi: 10.1111/ddg.14494 34164921

[18] Kridin K Bergman R. Assessment of the Prevalence of Mucosal Involvement in Bullous Pemphigoid. JAMA Dermatol (2019) 155(2):166–71. doi: 10.1001/jamadermatol.2018.5049 PMC6439539 30624571

[19] Muhammed N Korgaonkar S Pradhan V Khopkar US. A Cross-Sectional Study to Correlate Disease Severity in Bullous Pemphigoid Patients With Serum Levels of Autoantibodies Against BP180 and BP230. Indian Dermatol Online J (2021) 12(5):696–700. doi: 10.4103/idoj.IDOJ_813_20 34667755 PMC8456256

[20] Feng S Wu Q Jin P Lin L Zhou W Sang H. Serum Levels of Autoantibodies to BP180 Correlate With Disease Activity in Patients With Bullous Pemphigoid. Int J Dermatol (2008) 47(3):225–8. doi: 10.1111/j.1365-4632.2008.03473.x 18289320

[21] Cassano N Mastrandrea V Tampoia M Filotico R Vestita M Vena GA. Pemphigus Vulgaris With Circulating Anti-Desmoglein 3 and Anti-BP180 Antibodies: A Case Report and Brief Review of Cases With Coexistence of Pemphigus Vulgaris and Bullous Pemphigoid. J Biol Regul Homeost Agents (2009) 23(3):197–201. 19828097

[22] Fenner J Min MS Liu S Silverberg N. A Case of Neonatal Pemphigus Vulgaris With Co-Existing BP180 Autoantibodies. Pediatr Dermatol (2020) 37(1):241–3. doi: 10.1111/pde.14059 31774569

[23] Takahashi H Anzai H Suzuki Y Tanikawa A Amagai M Nishikawa T. Parallel Fluctuation of Anti-Desmoglein 3 and Anti-BP180 Autoantibody Titres in a Patient With Bullous Pemphigoid. Clin Exp Dermatol (2004) 29(6):608–11. doi: 10.1111/j.1365-2230.2004.01598.x 15550133

