# Supplementary material for: Case Report: Variety of Target Antigens During 1 Year Follow-Up of a Patient Initially Diagnosed With Bullous Pemphigoid
Source: Front Immunol. 2022 Jan 13;12:825226. doi: 10.3389/fimmu.2021.825226 (PMC8791857; doi:10.3389/fimmu.2021.825226)
Supplement: Supplementary file 1 [file Table_1.docx]

**Supplementary data**

**Table S1. Clinical features and steroid doses with disease course**

|  | **Clinical features** | **Methylprednisolone dose** |
| --- | --- | --- |
| Disease course before visit to our hospital | Blisters and erythema on the head, face and limbs, and itching had continued for half a year; and all symptoms got worse half a month ago. | - |
| Day 0 | Well-defined large erythema, various-sized tense blisters and erosions, and crusts on the erythema; these rashes mainly distributed on both the forearms and internal thighs; and no blister or erosion on the oral and genital mucosae. | 30 mg per day |
| Day 7 | No new blister or erythema, and no itching | 28 mg per day |
| Day 55 | No lesion, and no itching | 22 mg per day |
| Day 105 | No lesion, and no itching | 14 mg per day |
| Day 120 | No lesion, and no itching | 12 mg per day |
| Day 270 | One month ago, a few erythema and blisters re-appeared on the entire body, accompanied by itching; and one week ago, blisters and erosions firstly appeared in the oral cavity. At this time point, erythema, blisters, erosions and crusts widely distributed on the neck, trunk and limbs, accompanied by itching. | Stopped from Day 150 by the patient, and started with 40 mg per day from Day 270 |
| Day 279 | No new blister or erosion, most of erythema disappeared, itching relieved, and oral blisters and erosions disappeared. | 38 mg per day |
